# ADAM17-Mediated Reduction in CD14^++^CD16^+^ Monocytes *ex vivo* and Reduction in Intermediate Monocytes With Immune Paresis in Acute Pancreatitis and Acute Alcoholic Hepatitis

**DOI:** 10.3389/fimmu.2019.01902

**Published:** 2019-08-27

**Authors:** Kathryn Waller, Charlotte James, Anja de Jong, Laura Blackmore, Yun Ma, Andrew Stagg, David Kelsell, Michael O'Dwyer, Robert Hutchins, William Alazawi

**Affiliations:** ^1^Blizard Institute, Queen Mary University of London, London, United Kingdom; ^2^Institute of Liver Studies and Transplantation, King's College London, London, United Kingdom; ^3^St. Vincent's University Hospital, Dublin, Ireland; ^4^Hepatopancreaticobiliary Unit, Barts Health NHS Trust, London, United Kingdom

**Keywords:** monocytes, inflammation, ADAM17, infection, acute pancreatitis, acute alcoholic hepatitis

## Abstract

Impaired immune responses and increased susceptibility to infection characterize acute inflammatory conditions such as pancreatitis and alcoholic hepatitis and are major causes of morbidity and mortality. However, the mechanisms that drive this apparent immune paresis remain poorly understood. Monocytes mediate host responses to damage and pathogens in health and disease, and three subsets of monocytes have been defined based on CD14 and CD16 expression. We sought to determine the changes in monocyte subsets in acute pancreatitis (AP) and acute alcoholic hepatitis (AAH), together with functional consequences and mechanisms that underlie this change. Peripheral blood mononuclear cells (PBMCs) from patients with AP or AAH were compared with healthy controls. Monocyte subsets were defined by HLA-DR, CD14, and CD16 expression. Changes in surface and intracellular protein expression and phosphorylation were determined by flow cytometry. Phenotype and function were assessed following stimulation with lipopolysaccharide (LPS) or other agonists in the presence of specific inhibitors of TNFα and a disintegrin and metalloproteinase 17 (ADAM17). Patients with AP and AAH had reduced CD14^++^CD16^+^ intermediate monocytes compared to controls. Reduction of intermediate monocytes was recapitulated *ex vivo* by stimulating healthy control PBMCs with Toll-like receptor (TLR) agonists LPS, flagellin or polyinosilic:polycytidylic acid (poly I:C). Stimulation caused shedding of CD14 and CD16, which could be reversed using the ADAM17 inhibitor, TMI005 but not direct inhibitors of TNFα, a known ADAM17-target. Culturing PBMCs from healthy controls resulted in expansion of intermediate monocytes, which did not occur when LPS was in the culture medium. Cultured intermediate monocytes showed reduced expression of CX_3_CR1, CCR2, TLR4, and TLR5. We found reduced migratory responses, intracellular signaling and pro-inflammatory cytokine production, and increased expression of IL-10. Stimulation with TLR agonists results in ADAM17-mediated shedding of phenotypic markers from CD16^+^ monocytes, leading to apparent “loss” of intermediate monocytes. Reduction in CD14^++^CD16^−^ monocytes and increased CD14^++^CD16^+^ is associated with altered responses in functional assays *ex vivo*. Patients with AP and AAH had reduced proportions of CD14^++^CD16^+^ monocytes and reduced phosphorylation of NFκB and IL-6 production in response to bacterial LPS. Together, these processes may contribute to the susceptibility to infection observed in AP and AAH.

## Introduction

Impaired immune responses and increased susceptibility to bacterial infection characterize acute inflammatory conditions such as acute pancreatitis (AP) and alcoholic hepatitis (AAH) and are major causes of morbidity and mortality (1–5). However the mechanisms that drive this apparent immune paresis remain poorly understood. Monocytes play a pivotal role in the innate response to tissue damage and to pathogens, and studies have shown impaired function of the total monocyte population in patients with AP (6) and AAH (7). Monocytes are a heterogenous group of cells and our current understanding is that in humans, three subsets of monocytes can be distinguished based on the expression of CD14 and CD16. Classical monocytes (CD14^++^CD16^−^) are the most abundant in peripheral blood and are able to differentiate into monocyte-derived macrophages (8). Intermediate monocytes (CD14^++^CD16^+^) express high levels of HLA-DR and Toll-like receptors (TLRs) and are therefore considered to be an effector pro-inflammatory subset, contributing to antigen presentation and inflammatory cytokine production (8–10). The non-classical cells (CD14^+^CD16^+^) are small in number and their predominant role is believed to be patrol and surveillance of the vessel walls (11, 12). Plasticity between different subsets has been demonstrated. Classical monocytes have been shown to mature into intermediate and then into non-classical monocytes (13–16). This maturation sequence has been recapitulated in healthy volunteers following treatment with macrophage colony-stimulating factor (17), following LPS-induced monocytopenia with *in vivo* deuterium labeling and by fate mapping human classical monocytes that have been grafted into humanized mice (18).

Translational studies in humans show that systemic inflammatory diseases such as sepsis (10, 19), rheumatoid arthritis (20–22), Crohn's disease (23), and systemic lupus erythematosus (10, 24) are characterized by an increase in the proportion of CD16^+^ monocytes. Conversely, CD16^+^ monocytes are reduced in patients diagnosed with acute myocardial infarction at the time of admission although these cells significantly expanded over a week later (25).

AP and AAH are characterized by marked tissue and systemic inflammation that is believed to be a response to pathogen- and damage-associated molecular patterns (PAMPs and DAMPs) (26–29), some of which derive from the gut microbiome (30–33). In AP and AAH, classical inflammatory pathways are engaged, exemplified by PAMPS and DAMPs binding to pattern recognition receptors (PRRs). This leads to transcription factor activation and the expression of chemokines and cytokines. Post-translational processing by enzymes such as ADAM17 leads to cleavage and release of some of these mediators, such as TNFα. However, the relationship between these inflammatory phenomena and the relative proportions of monocyte subsets has received little attention until now. We hypothesize that exposure of monocytes to such inflammatory stimuli impacts monocyte plasticity and alters function. Here we find a reduction in intermediate monocytes in blood sampled from patients with AP and AAH compared to healthy volunteer controls. We find a similar reduction in intermediate monocytes when blood from healthy volunteers was stimulated with lipopolysaccharide (LPS) and other inflammatory agonists in an ADAM17-dependent mechanism. Moreover, while classical monocytes acquire surface markers of intermediate monocytes when cultured *ex vivo*, we found that these cells appear to be impaired. This work has implications for *in vitro* modeling of monocyte function and may contribute to the susceptibility to infection observed in patients with AP and AAH.

## Materials and Methods

### Patients

Patients and healthy control volunteers gave written informed consent and were recruited from Royal London and Kings College Hospitals, London UK. The study protocols were approved by the local research ethics committees (reference numbers 13/LO/0363, 15/LO/2127, and 15/SC/0224) and performed in compliance with the Declaration of Helsinki. We included patients with a clinical and biochemical or radiological diagnosis of mild acute pancreatitis (defined according to Atlanta criteria) (34) within 24 h of admission to the Royal London Hospital or severe acute alcoholic hepatitis (defined as Maddrey's Discriminant function ≥32) (35) within 24 h admission to Kings College Hospital. We excluded patients under 18 years, or those taking immunosuppression including methotrexate, biological therapy, ciclosporin, and tacrolimus.

### Cell Separation

Peripheral blood mononuclear cells (PBMCs) were separated by density gradient over Ficoll-Paque (GE Healthcare) as previously described (36). PBMCs from patients with AP and AAH and corresponding controls were cryopreserved in heat inactivated fetal bovine serum (HI-FCS [ThermoFisher Scientific]), 10% dimethyl sulfide (DMSO [Santa Cruz Biotechnology]). All other experiments used freshly isolated PBMCs with no cryopreservation. Viability of all PBMCs were assessed prior to all experiments using trypan blue (Sigma Aldrich) and only those with viability over 70% for cryopreserved samples and 90% for fresh samples, were included. Cells were seeded at 1 million per mL in RPMI 1640 containing L-glutamine (Lonza), penicillin/streptomycin (Sigma Aldrich), and 10% HI-FCS. PBMCs were stimulated with 20 ng/mL lipopolysaccharide from *Escherichia coli* O55:B5 (LPS [Sigma-Aldrich]), 10 μg/mL polyinosilic:polycytidylic acid (poly I:C [Sigma-Aldrich]), 100 ng/mL flagellin from *Salmonella typhimurium* (Source BioScience), 1 μg/mL high mobility group box 1 protein (HMGB1 [Sigma-Aldrich]), 1,000 units/mL interferon alpha (IFNα [Peprotech]) or 50 ng/mL interleukin 1 alpha (IL-1α [R&D Systems]) for 3 or 24 h in 12 well plates, with 1 mL per well. Monocytes were magnetically isolated using the Pan monocyte isolation kit (Miltenyi Biotec) according to the manufacturer's instructions.

### Characterizing Monocytes by Flow Cytometry

All fluorochrome-conjugated antibodies were purchased from Biolegend, unless otherwise stated. PBMCs were stained with antibodies including HLA-DR Phycoerythrin Cyanine 7 (PE-Cy7 [clone L243]), CD14 Pacific Blue (PB [clone ME52]), CD16 Alexa Fluor 647 (AF647 [clone 3G8]) TLR5 Fluorescein isothiocyanate (FITC [clone 85B152.5], Abcam), TLR4 Phycoerythrin (PE [clone HTA125]), CX3CR1 PE (clone 2A9-1), CCR2 PE (clone K036C2), CD80 PE (clone 2D10), CD86 FITC (clone BU63), CD115 Alexa Fluor 488 (AF488 [clone 9-4-D2-1E4]), and CD163 PE (clone GHI61). Viability of monocytes was assessed using Zombie Near Infrared (NIR) fixable viability kit. All flow cytometry experiments were acquired using a BD Canto II and analyzed using FlowJo v10.4.

### Tracking CD16+ Monocytes

Peripheral blood mononuclear cells were separated over Ficoll-Paque as above and the CD16^+^ monocytes were then magnetically isolated using a CD16^+^ monocyte isolation kit (Miltenyi Biotec) according to manufacturer's instructions. CD16^+^ monocytes were then washed in phosphate buffered solution (PBS) and incubated in PBS containing 2.5 μM Carboxyfluorescein succinimidyl ester (CFSE [Biolegend]) for 20 min at 37°C. Cells were then washed in RPMI 1640 containing penicillin/streptomycin, L-glutamine and 10% HI-FCS and added back into non-labeled PBMCs from the same donor. PBMCs were then seeded in 12 well plates in complete RPMI and incubated with or without 20 ng/mL LPS for 3 h prior to identification by flow cytometry, as above.

### Cell Viability

Viability of PBMCs was measured using a mammalian LIVE/DEAD™ viability/cytotoxicity kit (Invitrogen) as per the manufacturer's instructions. The plate-based assay measures a combination of elastase and calcein AM using fluorescence, detected by a spectrophotometer.

### ELISA

IL-6 and soluble CD14 were measured in PBMC supernatants using commercially available ELISA (R&D Systems). Soluble CD16 was measured as previously described (37). 96 well plates were pre-coated with 10 μg/mL anti-CD16 (clone SG8 [Biolegend]), recombinant CD16 (R&D Systems) was used to generate a standard curve and 0.5 μg/mL biotinylated anti-CD16 (Bio-Rad) was used as a detection antibody.

### Inhibitors

The small molecule TMI005 (Aprastat, Axon Medchem), a potent and selective dual inhibitor of ADAM17 and matrix metalloprotease was added to PBMCs (1 μg/mL) 45 min prior to the addition of LPS. To inhibit TNFα, the monoclonal antibody infliximab (gift from Dr Neil McCarthy, Blizard Institute, London, UK) was added to PBMCs (50 μg/mL) 45 min prior to the addition of LPS. The small molecule inhibitor of TNFα trimerization, SPD304 (Sigma Aldrich) was added to PBMCs (1 μM) for 9 h prior to the addition of LPS.

### Monocyte Function

Migration of monocytes was measured in response to MCP-1 (30 ng/mL [Sigma-Aldrich]) using transwell inserts (Corning). Migrated monocytes were identified in the lower chamber and counted by flow cytometry using flow-count fluorospheres (Beckman Coulter). *Ex vivo* IL-6, TNFα, and IL-10 production were measured by flow cytometry. Golgistop (BD Biosciences) was added to PBMCs prior to LPS stimulation for 4 h for IL-6 and TNFα measurements and overnight for IL-10. IL-6-PE (clone MQ2-13A5), TNFα-FITC (clone MAb11), and IL-10-PE (clone JES3-19F1) in permeabilization buffer (eBioscience) was used to determine IL-6, TNFα, and IL-10 expression, respectively. Phosphorylated NFκBp65 was measured by flow cytometry. PBMCs were stimulated with LPS (10 μg/mL) for 15 min at 37°C and fixed in 2% PFA followed by 90% methanol prior to staining with pS529 NFκBp65-PE (clone K10-895.12.50 BD Biosciences).

### Statistics

Statistical analyses were performed using GraphPad Prism 7.02. Normality was assessed using the Shapiro-Wilk normality test (38). Normally distributed data were analyzed by 2-tailed *t*-test. Mann-Whitney and Wilcoxon signed-rank tests were used to evaluate non-normally distributed data. Error bars in the figures indicate standard error of the mean. *P* < 0.05 was considered significant.

## Results

### Reduced Intermediate Monocytes in Acute Inflammatory States

**Figure 1:**
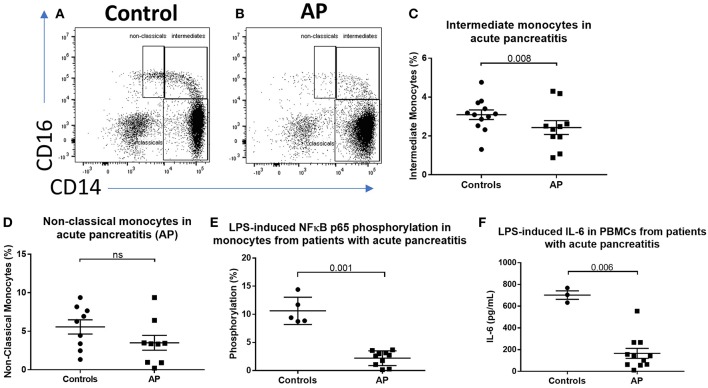
CD16^+^ monocytes are reduced in acute pancreatitits and in response to LPS. Representative flow cytometry plots showing monocyte subsets in **(A)** healthy controls or **(B)** patients with acute pancreatitis (AP). Monocytes were identified by forward and side scatter, HLA-DR positivity and classified according to the expression of CD14 and CD16. **(C)** Proportion of intermediate monocytes was significantly reduced in patients with AP compared with controls (*p* = 0.008). **(D)** Proportion of non-classical monocytes was reduced in patients with AP compared to controls [3.4 vs. 6.3% **(E)**]. Patients with AP had reduced levels of NFκBp65 phosphorylation at S529 in monocytes stimulated with LPS compared with controls (*p* = 0.001, percentage increase over unstimulated baseline). **(F)** Patients with AP had reduced LPS-induced IL-6 production from PBMCs compared to controls (*p* = 0.006).

To determine the effect of acute inflammation on monocyte phenotype, we studied blood sampled from patients with acute pancreatitis (AP) and compared the proportions of monocyte subsets with those from healthy controls. Eleven patients with mild AP admitted to hospital with biochemical or radiological evidence of AP were included (clinical details are shown in Supplemental Table 1). The percentages of intermediate (CD14^++^CD16^+^) and non-classical (CD14^+^CD16^+^) monocytes were lower in peripheral blood sampled from patients compared with controls (2.4 vs. 3.1% *p* = 0.008 and 3.4 vs. 6.3% *p* = 0.14, Figures 1A–D) Supplemental Figure 1 for gating strategy). There was evidence of altered immune function in peripheral blood mononuclear cells (PBMCs) sampled from AP patients with significantly lower levels of LPS-induced phosphorylated NF-kBp65 and *ex vivo* IL-6 production in patients compared to controls (Figures 1E,F). To determine whether the reduction of intermediate monocytes was only seen in pancreatic inflammation, we recruited eleven patients with severe acute alcoholic hepatitis (AAH); a florid inflammatory disease of the liver (clinical details are shown in Supplemental Table 2). We found a similar reduction in CD14^++^CD16^+^ intermediate and CD14^+^CD16^+^ non-classical monocytes in patients with AAH, 1 vs. 3.1%, *p* = 0.008 and 3.4 vs. 6.3% *p* = 0.033, Supplemental Figures 2A–C).

### ADAM17 Mediates LPS-induced Shedding of Intermediate Monocyte Phenotypic Surface Markers

**Figure 2:**
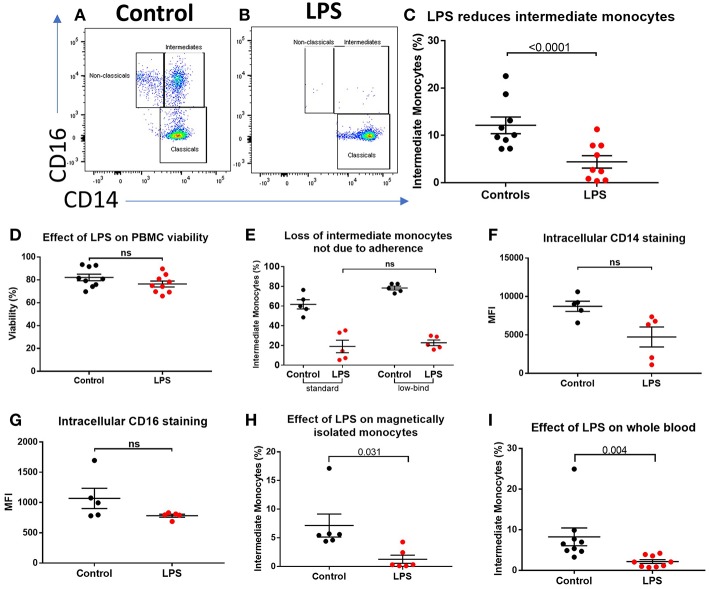
Apparent loss of intermediate monocytes in response to LPS stimulation *ex vivo*. **(A)** Representative flow cytometry plots showing monocyte subsets in healthy controls incubated for 3 h in medium **(B)** or LPS. **(C)** Intermediate monocytes as a proportion of all monocytes sampled from healthy controls were reduced after 3 h incubation with LPS compared with medium alone (*p* < 0.0001). **(D)** Viability of healthy control PBMCs sampled following 24 h incubation with medium and LPS by mammalian LIVE/DEAD™ viability/cytotoxicity kit (Invitrogen™), showing no increase in cell death with LPS (**ns** = not statistically significant). **(E)** No difference in proportion of intermediate monocytes from healthy controls following treatment with LPS when using standard tissue culture or ultra-low bind plates. **(F)** Intracellular staining for CD14 and **(G)** CD16 in monocytes was not increased following stimulation with LPS for 3 h. **(H)** Stimulation of magnetically-isolated healthy control monocytes with LPS for 3 h resulted in loss of cells from the intermediate monocyte gate (*p* = 0.031). **(I)** Intermediate monocytes were reduced in whole blood sampled from healthy donors incubated with LPS-stimulated for 3 h compared to medium alone (*p* = 0.004).

**Figure 3:**
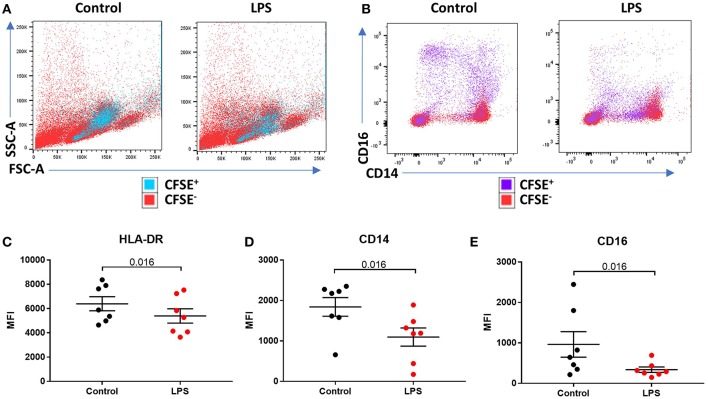
LPS-induced a reduction of phenotypic surface marker expression on intermediate monocytes. **(A,B)** Representative bivariate plots showing magnetically isolated, Carboxyfluorescein succinimidydl ester (CFSE) labeled, CD16+ monocytes incubated with or without LPS for 3 h. **(A)** Showing unchanged forward/side scatter and **(B)** marked reduction in CD16 and CD14 expression. **(C)** LPS induced a reduction in median intensity of **(C)** HLA-DR (*p* = 0.016), **(D)** CD14 (*p* = 0.016), and **(E)** CD16 (*p* = 0.016) on CFSE labeled CD16^+^ monocytes.

We sought to determine the underlying mechanism behind reduced intermediate monocytes in inflammatory disease. This unexpected inflammation-induced reduction in intermediate monocytes could be recapitulated *ex vivo* by stimulating PBMCs sampled from healthy controls with LPS (Figures 2A–C) or other TLR agonists; poly I:C and flagellin, but not IFNα, IL-1α, or HMGB1 (Supplemental Figure 3). Intermediate monocyte numbers were reduced in response to a range of lower concentrations of LPS down to 1 ng/mL (Supplemental Figure 4). The observed reduction in detected intermediate monocytes was not as a result of cell death (Figure 2D) and use of ultra-low bind plates did not affect this observation, excluding the possibility that activated intermediate monocytes simply adhere to plastic (Figure 2E). There was no increase in intracellular staining for CD14 or CD16 (and a statistically non-significant trend toward reduction) that might suggest internalization of the markers and hence inability to detect the intermediate monocytes that express them (Figures 2F,G). We also excluded the possibility that apparent loss of intermediate monocytes may be a product of the method used to isolate PBMCs (Figure 2H). Treating fresh whole blood with LPS led to a similar reduction in intermediate monocytes (Figure 2I). In order to track the fate of these cells following LPS stimulation, we magnetically sorted CD16^+^ monocytes from healthy controls and labeled them with CFSE (Figure 3A). Following stimulation with LPS, these CFSE^+^ cells that were previously in the intermediate monocyte gate persisted (Figure 3B) but had reduced HLA-DR (Figure 3C), CD14 (Figure 3D), and CD16 (Figure 3E) expression.

**Figure 4:**
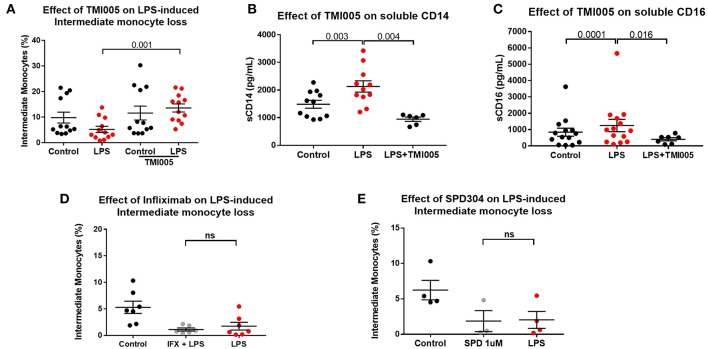
LPS-induced ADAM17-mediated CD14 and CD16 shedding from monocytes **(A)** Pre-treatment of PBMCs sampled from healthy controls with the ADAM17 inhibitor TMI005 (1 μg/mL) 45 min prior to incubation with LPS for 3 h prevented LPS-induced reduction of intermediate monocytes (*p* = 0.001). **(B)** Concentration of CD14 in supernatants from PBMCs increased following incubation with LPS for 3 h (*p* = 0.003) and was prevented by TMI005 (*p* = 0.004). **(C)** Concentration of CD16 in supernatants from PBMCs increased following incubation with LPS for 3 h (*p* = 0.0001) and was prevented by TMI005 (*p* = 0.016). Inhibition of TNFα with **(D)** Infliximab (50 μg/mL) or **(E)** SPD304 (1 μM) had no effect on LPS-induced apparent intermediate monocyte loss (**ns** = not statistically significant).

We therefore hypothesized that apparent “loss” of intermediate monocytes is, in fact, a result of loss of the markers used to detect them. ADAM17 (a metallopeptidase domain 17), also known as the tumor necrosis factor-alpha converting enzyme (TACE) is involved in ecto-domain shedding of TNFα in stimulated monocytes and CD16b shedding in activated NK cells (39–41). We therefore hypothesized a role for ADAM17 in LPS-induced apparent loss of intermediate monocyte and tested the effect of ADAM17 inhibition in LPS-stimulated PBMCs. Pre-treatment of PBMCs with TMI005 (a potent, selective ADAM17 inhibitor) prevented this apparent loss of intermediate monocyte in response to LPS (Figure 4A), poly I:C and a similar trend for flagellin (Supplemental Figure 5) and prevented LPS-induced shedding of CD14 (Figure 4B) and CD16 (Figure 4C). Inhibition of ADAM17 with TMI005 also prevented LPS-induced shedding of the colony stimulating factor 1 receptor (CD115) but not the scavenger receptor CD163 from monocytes (Supplemental Figure 6). We examined the possibility that inhibition of ADAM17 preserved the intermediate population through its known function in inhibiting TNFα release. Direct inhibition of TNFα inhibition with infliximab (a monoclonal antibody to TNFα) or SPD304 (a small molecule inhibitor of the TNFα trimer) did not have any effect in LPS-induced reduction in intermediate monocytes (Figures 4D,E).

### Phenotypic Changes Over Time Leads to Impaired Function of Monocytes *ex vivo*

**Figure 5:**
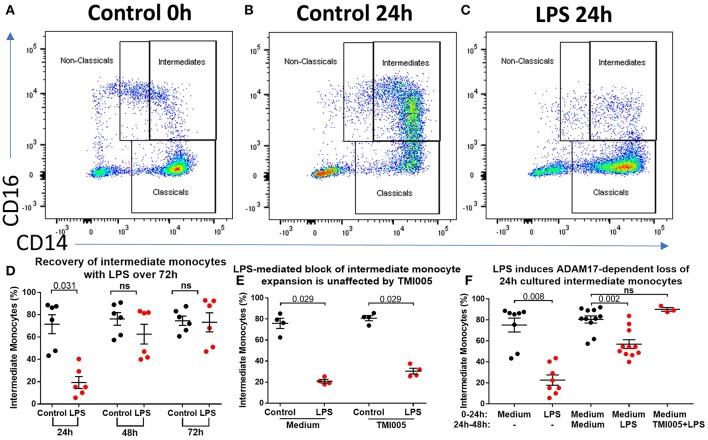
*Ex vivo* culture of monocytes leads to an altered phenotype. **(A)** Representative flow cytometry plots showing proportions of monocyte subsets from healthy controls **(B)** following incubation in culture for 24 h and **(C)** following incubation in culture in the presence of LPS for 24 h. **(D)** 24 h culture with LPS results in a smaller proportion of CD14^++^CD16^+^ monocytes (*p* = 0.003) but this population is restored with prolonged incubation in culture (**ns** = not statistically significant). **(E)** Addition of TMI005 (1 μg/mL) had no effect on LPS-mediated inhibition of monocyte maturation *ex vivo*, however **(F)** addition of LPS to PBMCs following 24 h incubation in unstimulated medium led to a reduction of intermediate monocytes in an ADAM17-dependent manner.

The data so far show that stimulation of PBMCs *ex vivo* results in a near total loss of cells from the intermediate monocyte gate in an ADAM17-dependent manner. However, in patients with AP and AAH, we saw a reduction, but not total loss, of cells from that gate. Therefore, we hypothesized that *in vivo*, apparent intermediate monocyte loss is concurrent with maturation of classical monocytes into intermediate monocytes as previously shown (18), and that this partially replenishes the CD14^++^CD16^+^ population. To study this further, we increased the incubation time from 3 to 24 h to study the effect of prolonged exposure to LPS/PAMPs. This resulted in marked expansion in CD14^++^CD16^+^ monocytes that did not occur when LPS was present in the culture medium (Figures 5A–C). However, prolonged incubation (for 48 or 72 h) overcomes this LPS-mediated block (Figure 5D) even when the culture medium was replenished with fresh medium containing LPS at 24 h and again at 48 h, excluding the possibility that the LPS was losing its effect through metabolism or degradation (Supplemental Figure 7). LPS-mediated block of CD14^++^CD16^+^ monocyte expansion over 24 h was not affected by ADAM17 inhibition with TMI005 (Figure 5E). However, when LPS was added to monocytes after they had been in culture for 24 h, there was a partial reduction of the CD14^++^CD16^+^ monocytes that could be prevented by addition of TMI005 (Figure 5F). This suggests that, at least with respect to ADAM17-dependent loss of intermediate monocyte phenotypic markers, these 24 h-cultured CD14^++^CD16^+^ monocytes behave like bone fide intermediate monocytes.

### Intermediate Monocytes Are Functionally Impaired Following Culture for 24 h

**Figure 6:**
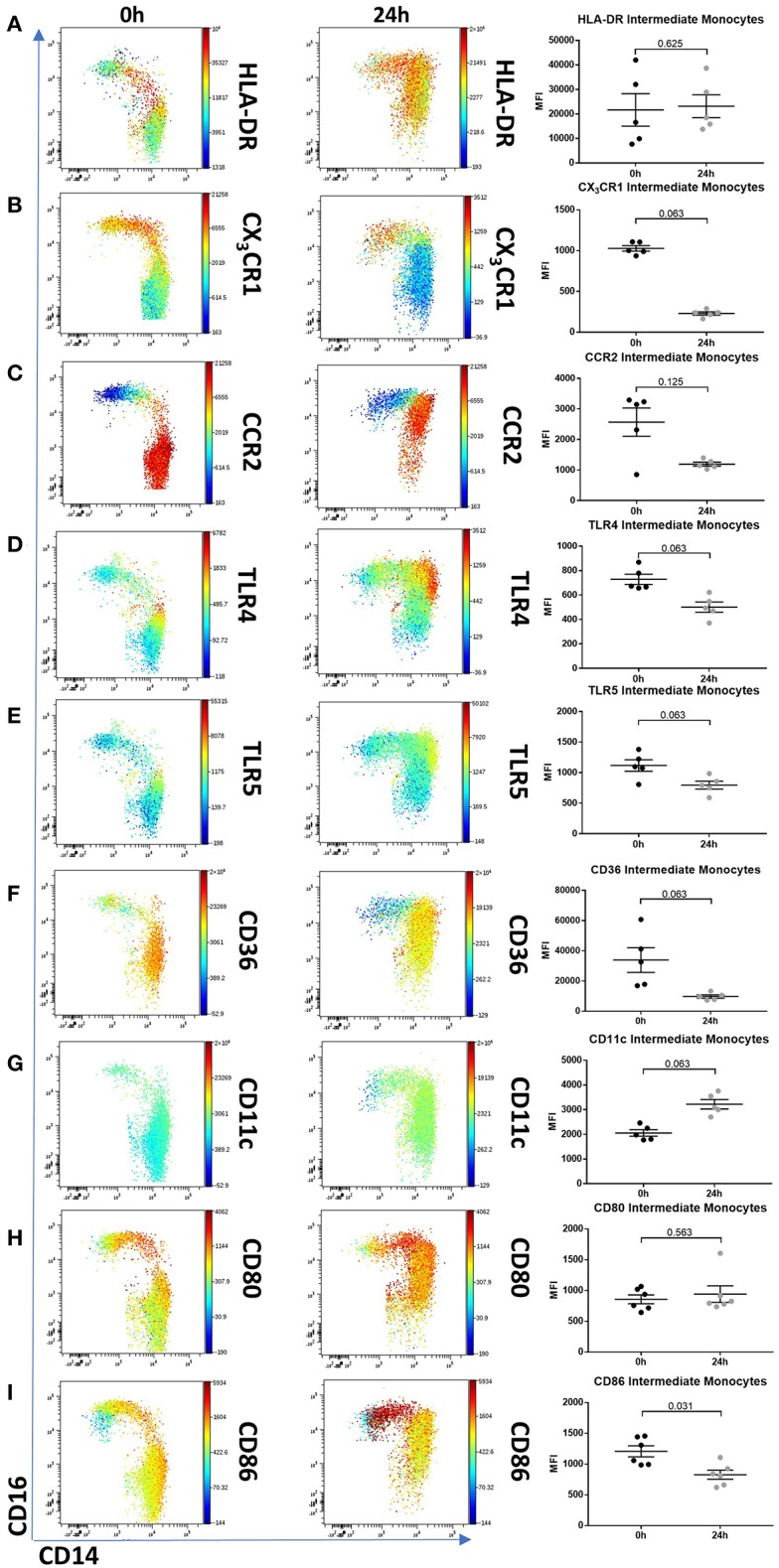
Intermediate monocytes have an altered phenotype after 24 h culture *ex vivo*. Representative viSNE plots (Cytobank, Santa Clara, USA) showing monocyte subsets illustrating expression of CD14 (X axis) and CD16 (Y axis). Median fluorescence intensity for each fluorochrome-conjugated antibody is represented according to the color bar shown. PBMCs sampled from healthy controls were incubated for 0 and 24 h in culture medium. **(A)** 24 h culture did not reduce intermediate monocytes HLA-DR expression but resulted in a reduced trend of **(B)** CX_3_CR1, **(C)** CCR2, **(D)** TLR4, **(E)** TLR5, and **(F)** CD36. Twenty-four hours culture increased intermediate monocyte expression of **(G)** CD11c, had no effect on **(H)** CD80 and a marginal reduction of **(I)** CD86. PBMCs sampled from 5 healthy controls were incubated for 0 and 24 h in culture medium and the expression for each fluorochrome-conjugated antibody on intermediate monocytes was illustrated in a dot plot.

**Figure 7:**
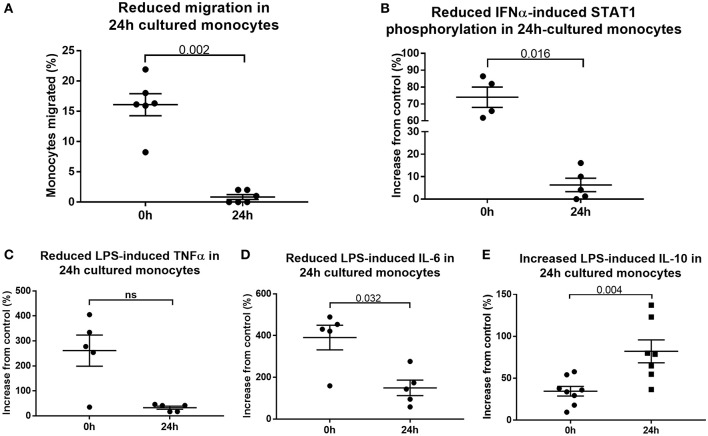
Cultured monocytes have reduced function. **(A)** Migration toward MCP-1 is impaired when PBMCs sampled from healthy controls were first incubated in medium for 24 h (*p* = 0.002). Monocytes retrieved from the lower chamber expressed as a percentage of the total added to the upper chamber of a transwell plate. Culture of PBMCs *ex vivo* impairs **(B)** IFNα-mediated STAT1 phosphorylation (*p* = 0.016), **(C)** LPS-induced TNFα (*p* = 0.056) and **(D)** LPS-induced IL-6 production (*p* = 0.032) **(E)** but increases LPS-induced IL-10 production (*p* = 0.004) as determined by flow cytometry.

To determine whether 24 h-cultured CD14^++^CD16^+^ monocytes behave like naturally-occurring intermediate monocytes in other ways, we examined the expression of a panel of cell surface markers known to distinguish monocyte subsets (8, 10, 18, 42) before and after 24 h culture (Figure 6, Supplemental Figure 8). Twenty-four hours culture resulted in reduced expression of CD86 (*p* = 0.031) and a trend toward reduction in expression of CD80, CX_3_CR1, CCR2, TLR4, TLR5, and CD36 on CD14^++^CD16^+^ cells (Figure 6); proteins involved in monocyte activation, survival, migration, pathogen activated molecular pattern (PAMP) signaling and scavenging, respectively. To determine whether this change in monocyte phenotype also affected function, we assessed cellular migration and cytokine production in response to LPS. Monocyte chemoattractant protein-1 (MCP-1) is potent chemoattractant (43, 44), and consistent with the downregulation of CCR2 (receptor for MCP-1), there was almost no migration of 24 h-cultured CD14^++^CD16^+^ monocytes in response to MCP-1 in a transwell migration assay (Figure 7A). Culture for 24 h also led to a reduction in IFNα-induced STAT1 phosphorylation (Figure 7B), a strong trend toward a reduction in LPS-induced TNFα, significantly reduced IL-6 production (Figures 7C,D), and increased LPS-induced expression of the anti-inflammatory cytokine IL-10 (Figure 7E).

## Discussion

Contrary to current dogma, we found a reduction in the proportion of intermediate monocytes in peripheral blood sampled from patients with two different acute inflammatory conditions, namely AP and AAH. While some have reported an increase in intermediate monocytes in sepsis (10, 19), others (18) demonstrate a reduction of CD14^+^CD16^++^ intermediate cells immediately following intravenous infusion of LPS in healthy volunteers. This discrepancy may be related to kinetics. Our blood samples were taken within 24 h of admission to hospital, albeit we cannot quantify the extent and duration of inflammation prior to hospitalization. Nevertheless, it is possible that sampling at a later stage of the illness may demonstrate recovery of intermediate monocytes as seen in the week following acute myocardial infarction (25). An early reduction in intermediate monocytes may be followed by expansion of cells bearing intermediate cell markers from the classical pool as we have described in the current study, albeit these cells that were grown in culture for 24 h appear to be functionally altered. In these cells, we showed reduced expression of TLR4 and TLR5 that are involved in sensing pathogen- and damage-associated molecular patterns as well as reduction in CX_3_CR1 that is involved in monocyte adhesion, survival, and recruitment (45, 46), CCR2 that mediates chemotaxis toward bacteria and necrotic cells, CD36, a receptor involved in scavenging debris and a reduction in the co-stimulatory molecule CD86 that mediates lymphocyte activation. Upregulation of IL-10 is further evidence of an immunosuppressive phenotype, and importantly also shows that this is an active process, or reflective of a physiological process of immune shut-down, rather than functional anergy. Indeed, LPS induces an anti-inflammatory state in monocytes from patients with acute alcoholic hepatitis, including increased IL-10 production, induction of PD1 and TIM3 and suppression of T cell responses (47). We hypothesize that this, together with the early loss of cells of CD14^++^CD16^+^ cells, may contribute to the increased susceptibility to infection which can complicate both AAH and AP (48–50). However, we acknowledge that *ex vivo* assays may not necessarily reflect the *in vivo* environment, and further whole animal or human studies are needed. We do not know whether the higher numbers of intermediate monocytes seen in chronic conditions (or that may be detectable later in the course of AAH or AP) are pro-inflammatory or whether they demonstrate the same altered function and non-response that we see *ex vivo*. Neonates and young children with sepsis following trauma have been shown to have increased proportions of CD16^+^ monocytes, and these monocytes also had reduced pro-inflammatory function; they were less able to phagocytose *E. coli*, produced less IFN gamma and had reduced expression of the activation marker CD86 (19).

While timing is undoubtedly important in this highly dynamic compartment of cells, the nature of the stimulus also plays a role. We found that stimulation for 3 h with each of LPS, poly I:C and flagellin, but not HMGB1, IFNα, or IL-1β caused reduction in intermediate monocytes. The mechanisms that underlie this agonist selectivity remain unclear, although TMI005 did reverse the reduction where it was observed, irrespective of the agonist. LPS, poly I:C and flagellin mainly activate TLR4, TLR3, and TLR5, respectively; whereas HMGB1 (which did not induce the same phenotypic changes in intermediate monocytes) can activate both TLR4 (51–54) and TLR5 (55), suggesting that signaling factors downstream of the TLR determine ADAM17 activation. Our results suggest that CD14 and CD16 are not internalized and also indicate a reduction of intracellular staining for these markers, although not statistically significant. One explanation is that permeabilisation of the cells does not preclude binding of antibodies to cell surface markers. Therefore, the reduction observed, if real, may simply reflect reduction in total intracellular and surface staining. An alternative explanation is that there is a reduction in the total pool of CD14 and CD16 across all cellular compartments, hence the reduction observed. The key conclusion, however, is that intracellular levels do not increase following stimulation. Other possibilities that may explain the apparent loss of intermediate monocytes include sequestration in the vasculature or inflamed tissues or cell death.

We find parallels between reduced numbers of CD14^++^CD16^+^ intermediate monocytes in patients with AP and AAH, and reduced numbers CD14^++^CD16^+^ cells following *ex vivo* treatment of PBMCs with different inflammatory mediators. It has previously been shown that ADAM17 mRNA is upregulated in the pancreas from patients with pancreatitis (56) and in PBMCs and liver tissue from patients with chronically inflamed hepatic iron-overload compared to patients without iron-overload (57). Patients with AP (58) and AAH (59) have elevated plasma levels of CD14. LPS levels are elevated in both AAH (206.9 pg/mL ± 174.9 pg/mL) (60) and animal models of AP (61, 62). However, given that reduction of CD14^++^CD16^+^ cells also follows flagellin and poly I:C stimulation, LPS is unlikely to be the only mediator of such a process in patients with complex inflammatory disease. We were unable to measure levels of ADAM17, LPS, CD14, or CD16 in patient plasma, due to lack of residual material. Nevertheless, neither these published observations nor further similar measurement in our patients would be sufficient to prove that the same mechanism we observe *ex vivo* is responsible for reduction of intermediate monocytes *in vivo*. Due to a lack of residual samples these experiments were not completed. However, this hypothesis does warrant further examination in the form of a human intervention study.

It might even be attractive to consider inhibition of ADAM17 as a therapeutic approach in patients with these conditions. However, while our data raise the possibility that ADAM17 may mediate part of the increased susceptibility to infection in patients with AP and AAH, they cannot be taken to imply a role for ADAM17 in the primary pathogenesis of these conditions. ADAM17 knockout mice are not viable, but a genetically engineered mouse that expresses very low levels of ADAM17 in all tissues does exist and shows increased susceptibility to dextran sulfate sodium-induced colitis (63). Conversely, mice with leucocyte-specific deficiency in ADAM17 are viable and are less susceptible to *E. coli*-mediated peritoneal sepsis (64). The specific small molecule ADAM17 inhibitor that we used in the current study, TMI005 did not show any efficacy in a phase II trial of rheumatoid arthritis but was well-tolerated (65). To our knowledge, there have been no studies of ADAM17 inhibition as a strategy to reduce the risk of infection in patients with acute inflammatory conditions such as AP or AAH. The variability in responses to ADAM17-inhibition is due, at least in part, to the multiple downstream targets of ADAM17 (66). The best-known of these targets is TNFα, but we did not see an effect on intermediate monocyte proportions with inhibition of TNFα. Nevertheless, infliximab, a monoclonal antibody targeted against TNFα, has shown improvements in rats with AP (67) and a clinical trial of infliximab in AP is due to start recruiting soon (https://clinicaltrials.gov/ct2/show/NCT03684278).

Our data offer new insight into the plasticity and function of monocyte subsets. Phenotypic classification based on CD14 and CD16 expression alone appears insufficient to describe the maturation process nor to define functional status. The markers and functions we have studied here identify functional differences between native and 24 h-cultured intermediate monocytes, and the true extent of these differences are likely to extend much further into processes we have not assessed, for example phagocytosis or antigen presentation.

There is a major unmet clinical need for therapies that target the acute inflammatory response in important inflammatory diseases such as AP and AAH and the resultant susceptibility to infection seen in these patients. Understanding processes such as reduction in effector intermediate monocytes and expansion of functionally inert CD14^++^CD16^+^ monocytes will advance the development of such therapeutics.

## Data Availability

All datasets generated for this study are included in the manuscript and/or the Supplementary Files.

## Ethics Statement

Patients and healthy control volunteers gave written informed consent and were recruited from Royal London and Kings College Hospitals, London UK. The study protocols were approved by the local research ethics committees (reference numbers 13/LO/0363, 15/LO/2127, and 15/SC/0224) and performed in compliance with the Declaration of Helsinki.

## Author Contributions

KW, CJ, and AdJ performed the experiments. KW analyzed and interpreted the data and wrote the manuscript. RH identified patients and supervised the clinical data collection. CJ and LB recruited the patients and prepared the samples. DK contributed to the experimental design. AS, MO'D, YM, and RH provided critical appraisal of the project and manuscript. WA conceived the study, interpreted the data, and wrote the manuscript. All authors approved the final version.

### Conflict of Interest Statement

The authors declare that the research was conducted in the absence of any commercial or financial relationships that could be construed as a potential conflict of interest.

## Supplementary Material

Supplemental Figure 1 Flow cytometry gating strategy. **(A)** Monocytes were first identified by forward and side scatter, **(B)** doublets were then discriminated using forward scatter height and area. **(C)** dead cells were discriminated using Near-Infrared (NIR) zombie viability dye and **(D)** finally HLA-DR positive cells were selected. **(E–G)** Monocyte subsets were characterized by CD14 and CD16 expression which was determined using concentration-matched isotype controls for Pacific Blue and Alexa Fluor 647, respectively.

Supplemental Figure 2 CD16^+^ monocytes are reduced in acute alcoholic hepatitis. **(A)** Representative FACS plots showing monocyte subsets in healthy controls or patients with AAH. Monocytes were identified by forward and side scatter, HLA-DR positivity and classified according to the expression of CD14 and CD16. **(B)** Proportion of intermediate monocytes were significantly reduced in AAH patients compared with controls (*p* = 0.008; controls are the same as in Figure 1). **(C)** Proportion of non-classical monocytes were significantly reduced in AAH patients compared with controls (*p* = 0.033).

Supplemental Figure 3 Proportions of intermediate monocytes are reduced in response to LPS, poly I:C and flagellin. PBMCs sampled from healthy controls (*n* = 9; *n* = 7 for flagellin) were stimulated for 3 h with LPS (20 ng/mL), poly I:C (10 μg/mL), flagellin (100 ng/mL), IFNα (1,000 U/mL), IL-1α (50 ng/ml), and HMGB1 (1 μg/mL) and intermediate monocytes were determined using flow cytometry. Cells in the intermediate monocyte gate were reduced when stimulated with LPS (*p* = 0.004), poly I:C (*p* = 0.027), and flagellin (*p* = 0.016).

Supplemental Figure 4 LPS induces a reduction of CD14^+^ and CD16^+^ monocytes in a concentration-dependent manner.Representative flow cytometry plots showing monocyte subsets from healthy controls, following incubation with 1, 10, 20, and 100 ng/mL LPS for 3 h.

Supplemental Figure 5 Inhibiting ADAM17 prevents stimulation-induced loss of cells with intermediate monocyte phenotype. PBMCs sampled from healthy controls (*n* = 5) were cultured with 1 μg/mL TMI005 for 45 min prior to stimulation with LPS (20 ng/mL), poly I:C (10 μg/mL) or flagellin (100 ng/mL) for 3 h. Incubation with TMI005 prevented LPS and poly I:C induced reduction of cells in the intermediate monocyte gate, with a similar trend toward significance for flagellin (**ns** = not statistically significant).

Supplemental Figure 6 CD115 and CD163 surface expression are ADAM17-mediated on monocytes. **(A)** Treatment of PBMCs sampled from healthy controls with LPS for 3 h induced a reduction in surface expression of CD115 on monocytes (*p* = 0.003) which was prevented by pre-treatment with the ADAM17 inhibitor TMI005 (1 μg/mL) 45 min prior to incubation with LPS. ADAM17 inhibition prevented LPS-induced reduction in surface expression of CD115 (*p* = 0.008) but not **(B)** CD163 (**ns** = not statistically significant).

Supplemental Figure 7 Intermediate monocytes recover with prolonged stimulation with replenished LPS. PBMCs sampled from 8 healthy controls were incubated in medium and medium containing LPS for 24, 48, or 72 h and intermediate monocytes were determined using flow cytometry. PBMCs that were incubated for over 24 h had media and LPS replenished every 24 h. Replenishment with fresh medium, with or without LPS, did not prevent the recovery of cells in the intermediate monocyte gate after 72 h (**ns** = not statistically significant).

Supplemental Figure 8 Monocyte subsets have an altered phenotype after 24 h culture. PBMCs sampled from healthy controls (*n* = 5) were incubated for 0 and 24 h in culture medium and the median fluorescence intensities of **(A)** HLA-DR, **(B)** CX_3_CR1, **(C)** CCR2, **(D)** TLR4, **(E)** TLR5, **(F)** CD36, **(G)** CD11c, **(H)** CD80, and **(I)** CD86 on monocyte subsets were evaluated using flow cytometry.

Supplemental Table 1 Clinical characteristics of patients with acute pancreatitis. Patients 2 and 8 were diagnosed radiologically. WCC, white cell count; Alc, alcohol; Tr, Trauma; Ga, gallstones; U, unknown.

Supplemental Table 2 Clinical characteristics of patients with acute alcoholic hepatitis. WCC, white cell count; AST, aspartate aminotransferase; INR, international normalized ratio; DF, discriminant function; MELD, Model for end-stage liver disease; CP, Child-Pugh.
