# Supplementary figures and images for: ADAM17-Mediated Reduction in CD14++CD16+ Monocytes ex vivo and Reduction in Intermediate Monocytes With Immune Paresis in Acute Pancreatitis and Acute Alcoholic Hepatitis
Source: Front Immunol. 2019 Aug 27;10:1902. doi: 10.3389/fimmu.2019.01902 (PMC6718469; doi:10.3389/fimmu.2019.01902)

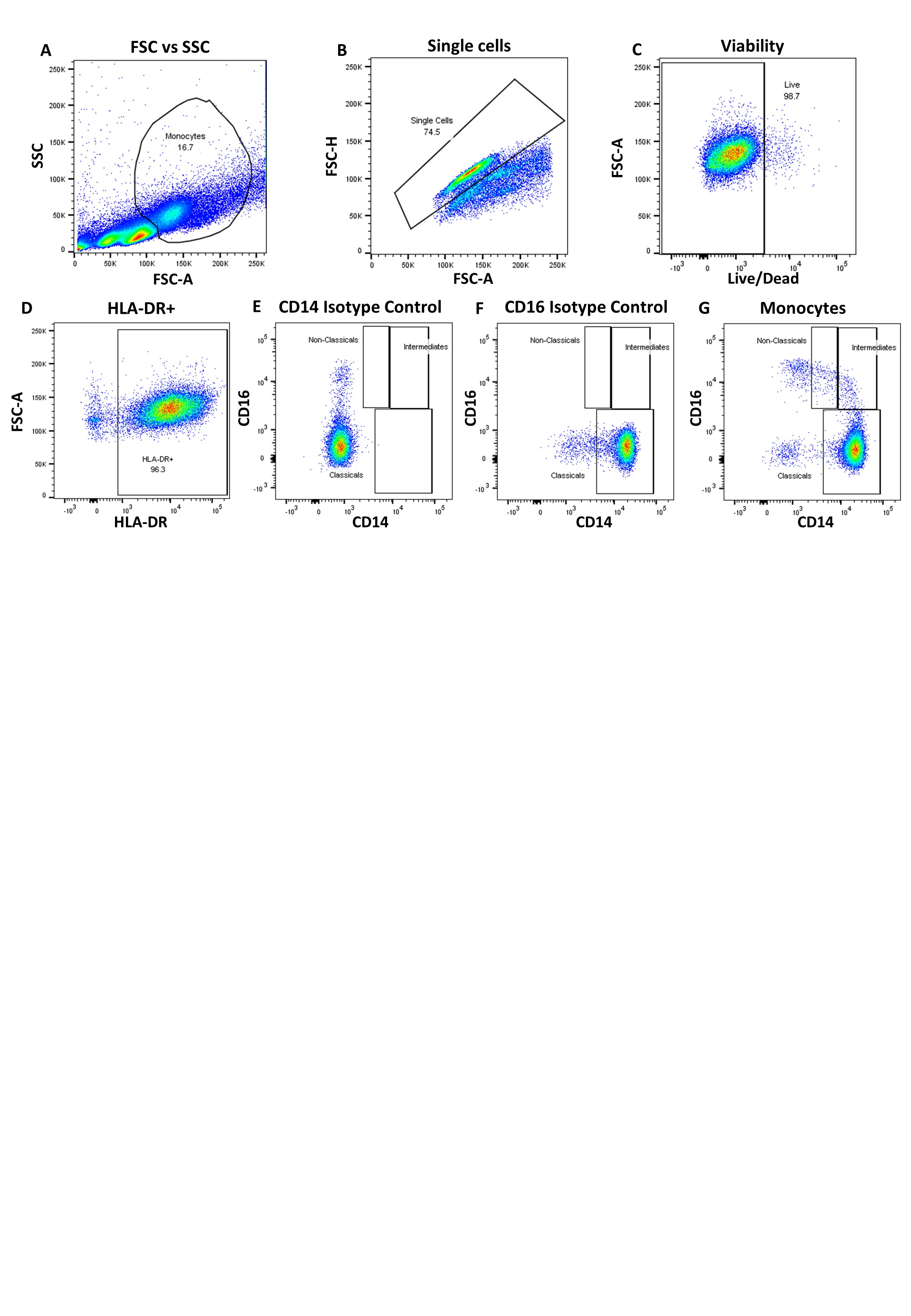

Supplement: Supplemental Figure 1 — Flow cytometry gating strategy. (A) Monocytes were first identified by forward and side scatter, (B) doublets were then discriminated using forward scatter height and area. (C) dead cells were discriminated using Near-Infrared (NIR) zombie viability dye and (D) finally HLA-DR positive cells were selected. (E–G) Monocyte subsets were characterized by CD14 and CD16 expression which was determined using concentration-matched isotype controls for Pacific Blue and Alexa Fluor 647, respectively. [file Image_1.JPEG]

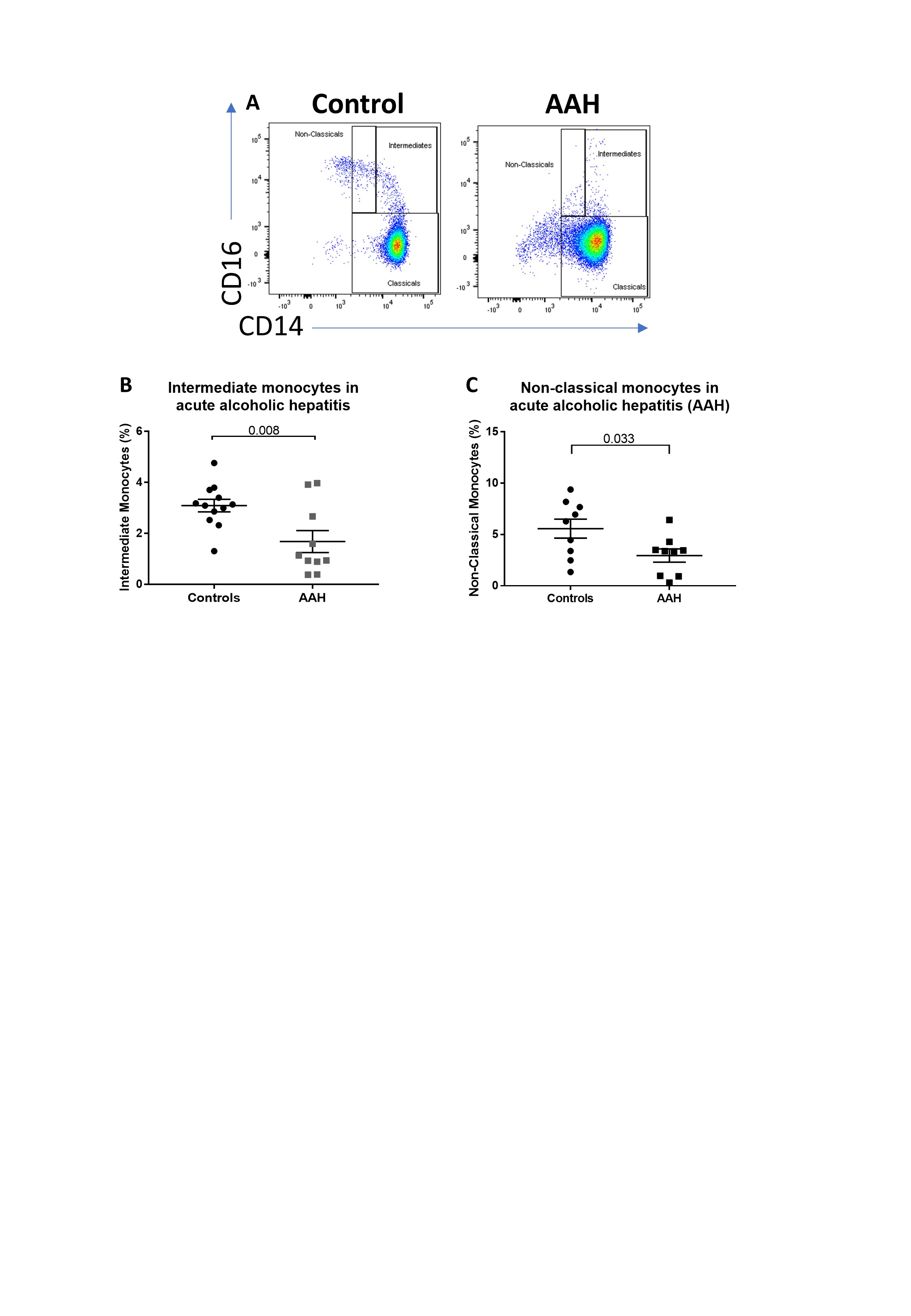

Supplement: Supplemental Figure 2 — CD16+ monocytes are reduced in acute alcoholic hepatitis. (A) Representative FACS plots showing monocyte subsets in healthy controls or patients with AAH. Monocytes were identified by forward and side scatter, HLA-DR positivity and classified according to the expression of CD14 and CD16. (B) Proportion of intermediate monocytes were significantly reduced in AAH patients compared with controls (p = 0.008; controls are the same as in Figure 1). (C) Proportion of non-classical monocytes were significantly reduced in AAH patients compared with controls (p = 0.033). [file Image_2.JPEG]

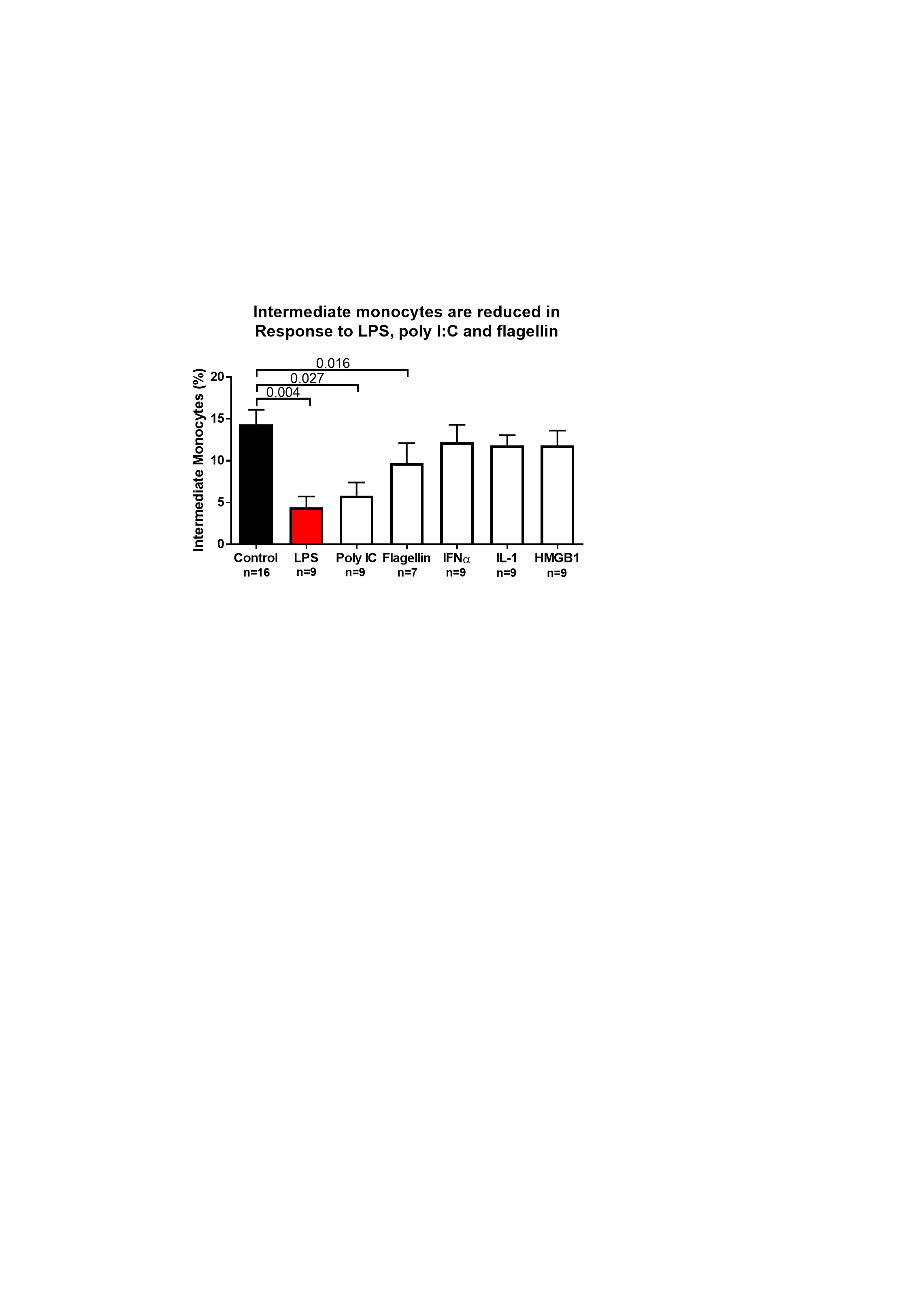

Supplement: Supplemental Figure 3 — Proportions of intermediate monocytes are reduced in response to LPS, poly I:C and flagellin. PBMCs sampled from healthy controls (n = 9; n = 7 for flagellin) were stimulated for 3 h with LPS (20 ng/mL), poly I:C (10 μg/mL), flagellin (100 ng/mL), IFNα (1,000 U/mL), IL-1α (50 ng/ml), and HMGB1 (1 μg/mL) and intermediate monocytes were determined using flow cytometry. Cells in the intermediate monocyte gate were reduced when stimulated with LPS (p = 0.004), poly I:C (p = 0.027), and flagellin (p = 0.016). [file Image_3.JPEG]

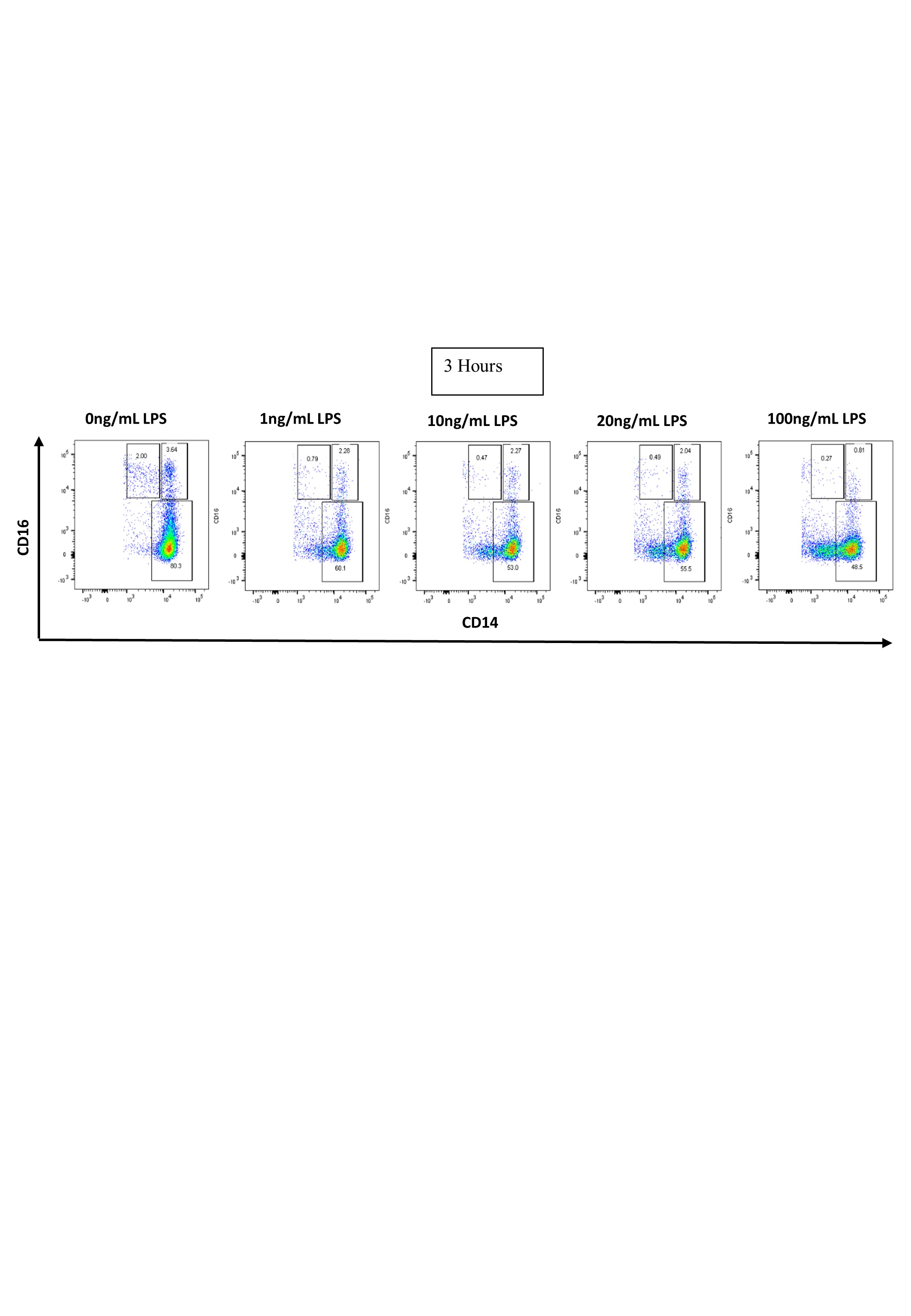

Supplement: Supplemental Figure 4 — LPS induces a reduction of CD14+ and CD16+ monocytes in a concentration-dependent manner.Representative flow cytometry plots showing monocyte subsets from healthy controls, following incubation with 1, 10, 20, and 100 ng/mL LPS for 3 h. [file Image_4.JPEG]

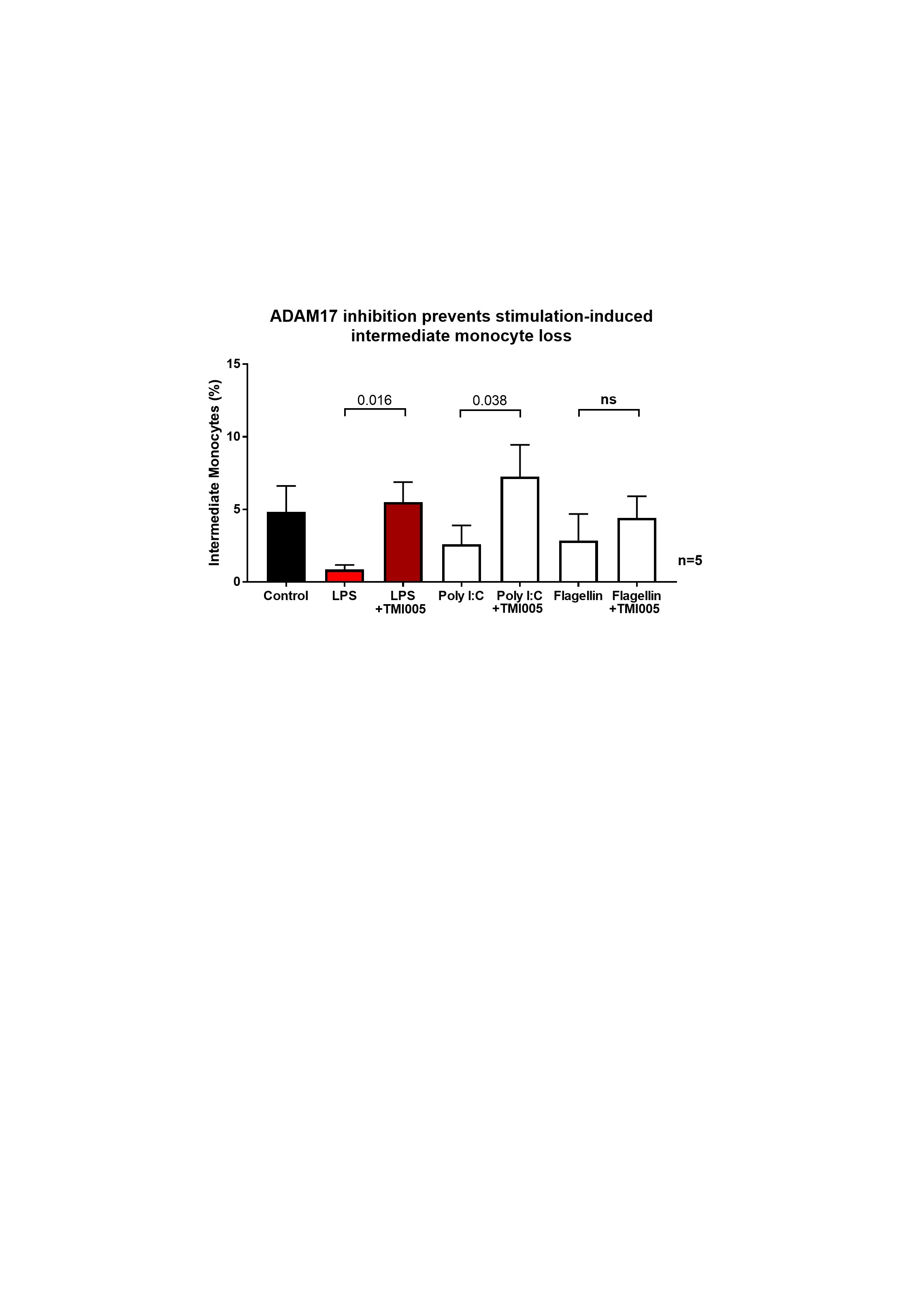

Supplement: Supplemental Figure 5 — Inhibiting ADAM17 prevents stimulation-induced loss of cells with intermediate monocyte phenotype. PBMCs sampled from healthy controls (n = 5) were cultured with 1 μg/mL TMI005 for 45 min prior to stimulation with LPS (20 ng/mL), poly I:C (10 μg/mL) or flagellin (100 ng/mL) for 3 h. Incubation with TMI005 prevented LPS and poly I:C induced reduction of cells in the intermediate monocyte gate, with a similar trend toward significance for flagellin (ns = not statistically significant). [file Image_5.JPEG]

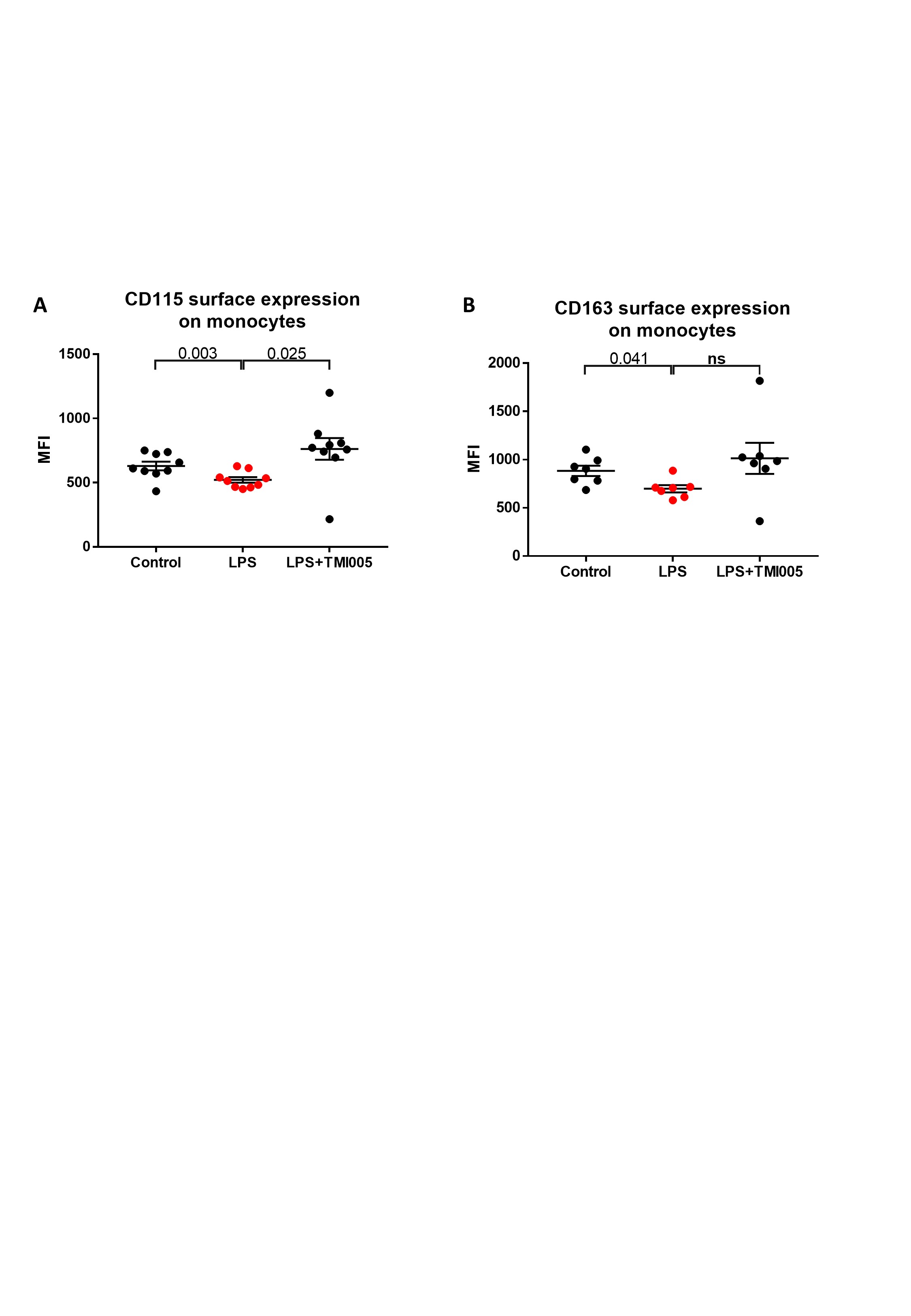

Supplement: Supplemental Figure 6 — CD115 and CD163 surface expression are ADAM17-mediated on monocytes. (A) Treatment of PBMCs sampled from healthy controls with LPS for 3 h induced a reduction in surface expression of CD115 on monocytes (p = 0.003) which was prevented by pre-treatment with the ADAM17 inhibitor TMI005 (1 μg/mL) 45 min prior to incubation with LPS. ADAM17 inhibition prevented LPS-induced reduction in surface expression of CD115 (p = 0.008) but not (B) CD163 (ns = not statistically significant). [file Image_6.JPEG]

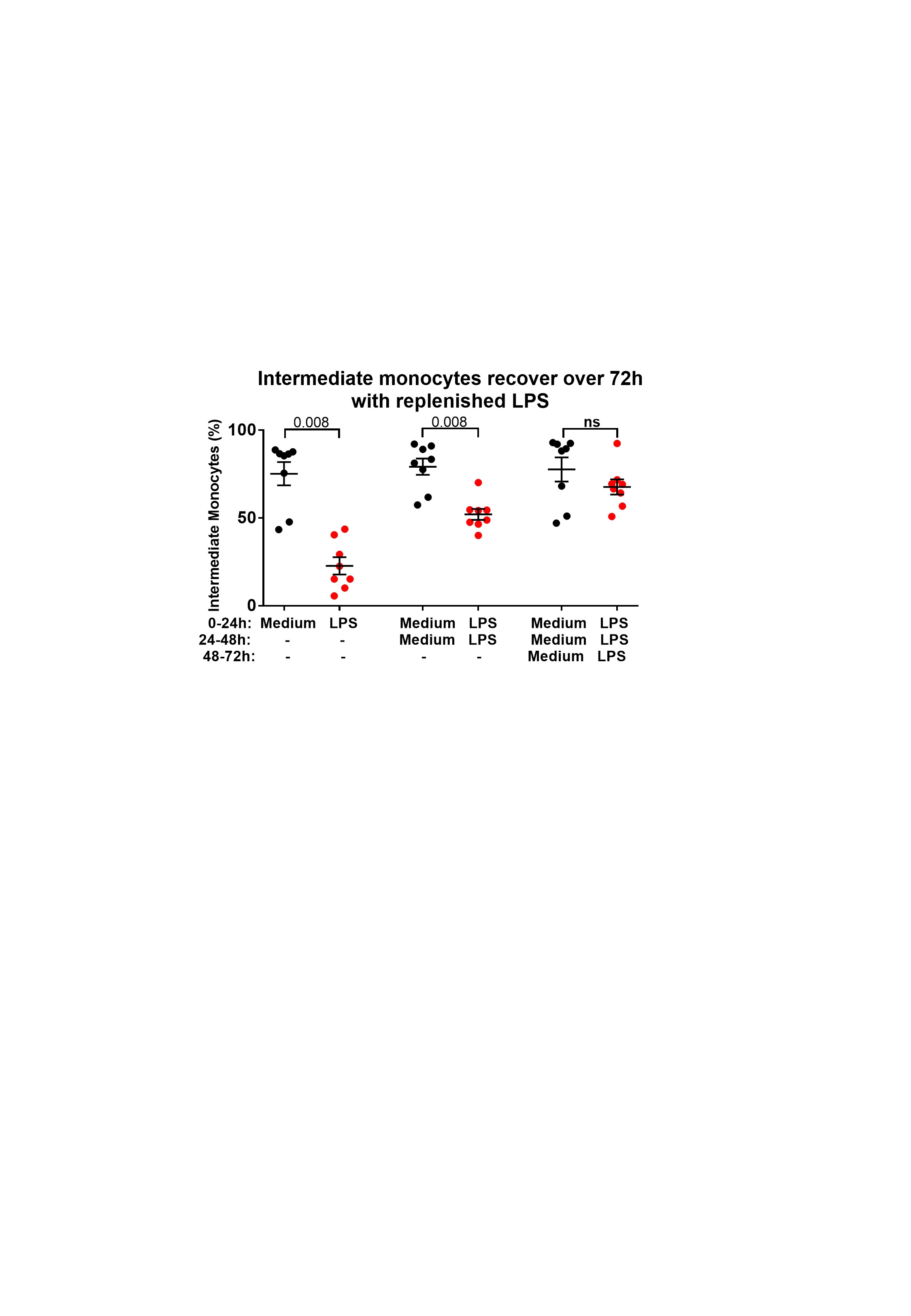

Supplement: Supplemental Figure 7 — Intermediate monocytes recover with prolonged stimulation with replenished LPS. PBMCs sampled from 8 healthy controls were incubated in medium and medium containing LPS for 24, 48, or 72 h and intermediate monocytes were determined using flow cytometry. PBMCs that were incubated for over 24 h had media and LPS replenished every 24 h. Replenishment with fresh medium, with or without LPS, did not prevent the recovery of cells in the intermediate monocyte gate after 72 h (ns = not statistically significant). [file Image_7.JPEG]

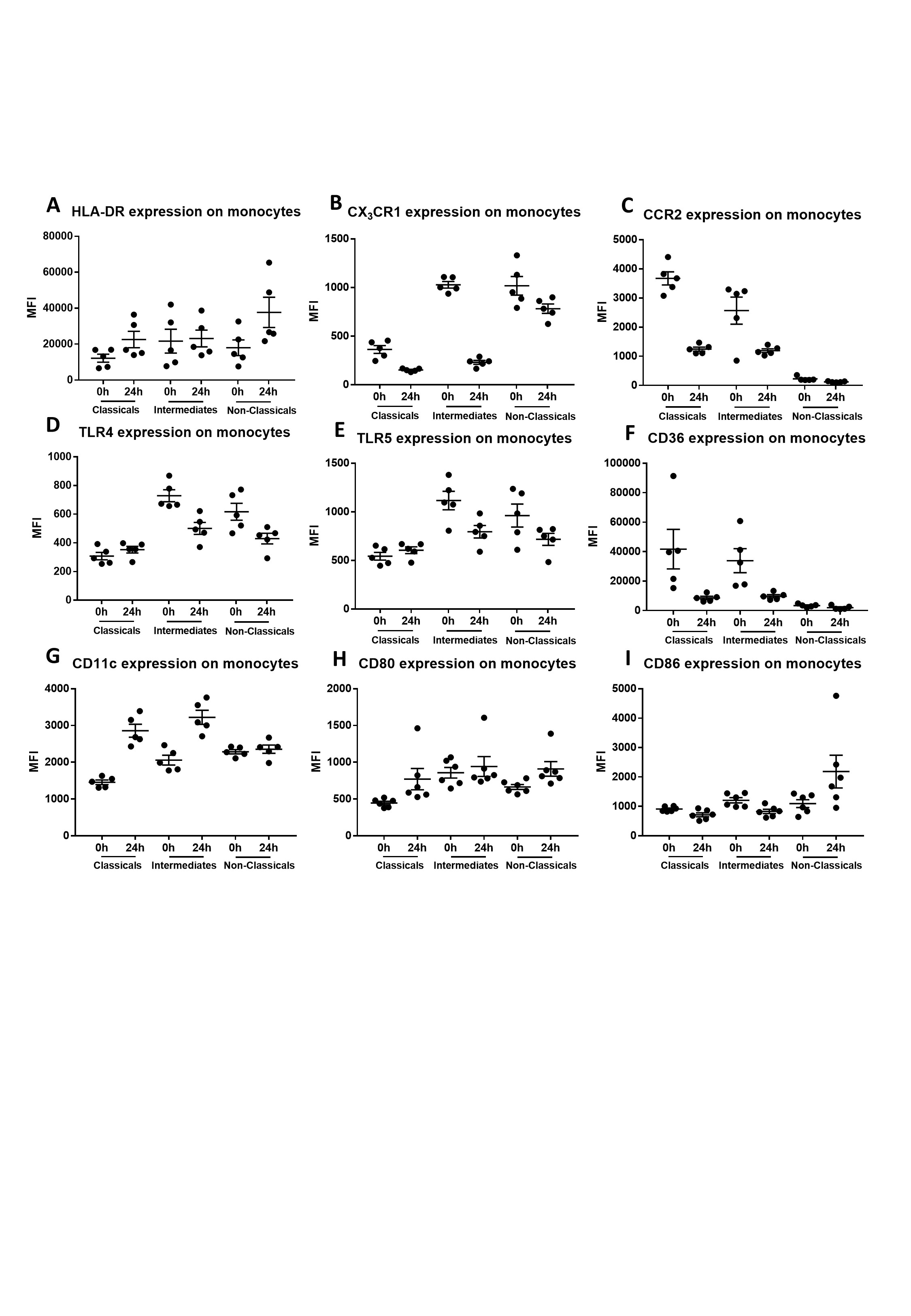

Supplement: Supplemental Figure 8 — Monocyte subsets have an altered phenotype after 24 h culture. PBMCs sampled from healthy controls (n = 5) were incubated for 0 and 24 h in culture medium and the median fluorescence intensities of (A) HLA-DR, (B) CX3CR1, (C) CCR2, (D) TLR4, (E) TLR5, (F) CD36, (G) CD11c, (H) CD80, and (I) CD86 on monocyte subsets were evaluated using flow cytometry. [file Image_8.JPEG]
