# Supplementary material for: ADAM17-Mediated Reduction in CD14++CD16+ Monocytes ex vivo and Reduction in Intermediate Monocytes With Immune Paresis in Acute Pancreatitis and Acute Alcoholic Hepatitis
Source: Front Immunol. 2019 Aug 27;10:1902. doi: 10.3389/fimmu.2019.01902 (PMC6718469; doi:10.3389/fimmu.2019.01902)
Supplement: Supplemental Table 1 — Clinical characteristics of patients with acute pancreatitis. Patients 2 and 8 were diagnosed radiologically. WCC, white cell count; Alc, alcohol; Tr, Trauma; Ga, gallstones; U, unknown. [file Table_1.DOCX]

| **Patient Number** | **1** | **2** | **3** | **4** | **5** | **6** | **7** | **8** | **9** | **10** | **11** | **Mean** |
| --- | --- | --- | --- | --- | --- | --- | --- | --- | --- | --- | --- | --- |
| **Age (y)** | 50 | 33 | 19 | 44 | 52 | 59 | 33 | 48 | 55 | 53 | 79 | **47.7** |
| **Haemoglobin (g/l)** | 137 | 112 | 159 | 108 | 133 | 130 | 145 | 122 | 131 | 186 | 130 | **135.7** |
| **WCC x10^9/l** | 6.5 | 11.5 | 6.5 | 7.5 | 13.2 | 8 | 11.3 | 12.6 | 5.4 | 19.4 | 12.4 | **10.4** |
| **Neutrophils x10^9/l** | 3.5 | 5.7 | 4.6 | 5.3 | 11.5 | 6.2 | 8.5 | 8.2 | 3.1 | 16.9 | 10.8 | **7.7** |
| **Monocytes x10^9/l** | 0.4 | 1.4 | 0.5 | 0.3 | 1.1 | 0.6 | 0.8 | 0.6 | 0.3 | 1.2 | 0.7 | **0.7** |
| **Albumin g/l** | 42 | 30 | 47 | 33 | 49 | 44 | 46 | 42 | 42 | 32 | 32 | **39.9** |
| **CRP mg/l** | 5 | 248 | 5 | 14 | 41 | 5 | 24 | 50 | 7 | 393 | 363 | **105.0** |
| **Amylase** | 552 | 27* | 486 | 239 | 462 | 1366 | 236 | 46* | 1086 | 470 | 1157 | **672.7** |
| **Platelets x10^9/l** | 433 | 163 | 203 | 108 | 229 | 64 | 349 | 320 | 207 | 160 | 189 | **220.5** |
| **Aetiology** | Alc | Tr | Ga | Alc | Ga | U | Alc | Alc | Ga | Ga | Ga |  |

**Supplemental Table 1. Clinical characteristics of patients with acute pancreatitis.** Patients 2 and 8 were diagnosed radiologically. WCC – white cell count. Alc – alcohol, Tr – Trauma, Ga – gallstones, U – unknown
