# Supplementary material for: ADAM17-Mediated Reduction in CD14++CD16+ Monocytes ex vivo and Reduction in Intermediate Monocytes With Immune Paresis in Acute Pancreatitis and Acute Alcoholic Hepatitis
Source: Front Immunol. 2019 Aug 27;10:1902. doi: 10.3389/fimmu.2019.01902 (PMC6718469; doi:10.3389/fimmu.2019.01902)
Supplement: Supplemental Table 2 — Clinical characteristics of patients with acute alcoholic hepatitis. WCC, white cell count; AST, aspartate aminotransferase; INR, international normalized ratio; DF, discriminant function; MELD, Model for end-stage liver disease; CP, Child-Pugh. [file Table_2.DOCX]

| **Patient Number** | **1** | **2** | **3** | **4** | **5** | **6** | **7** | **8** | **9** | **10** | **11** | **Mean** |
| --- | --- | --- | --- | --- | --- | --- | --- | --- | --- | --- | --- | --- |
| **Age (y)** | 64 | 49 | 39 | 63 | 25 | 49 | 43 | 38 | 48 | 31 | 41 | 44.5 |
| **Haemoglobin (g/l)** | 13.4 | 8.2 | 9.2 | 13.3 | 9 | 10.3 | 7.4 | 9.1 | 8.8 | 8.8 | 9.3 | 9.1 |
| **WCC x10^9/l** | 5.4 | 12 | 5.6 | 8.3 | 5 | 10.9 | 12.1 | 14.3 | 26.9 | 2.5 | 5.7 | 9.9 |
| **Neutrophils x10^9/l** | 3.2 | 10.1 | 3.3 | 5.5 | 3 | 9.5 | 9.5 | 11.8 | 23.5 | 1.5 | 4.4 | 7.75 |
| **Platelets x10^9/l** | 67 | 146 | 76 | 130 | 228 | 111 | 168 | 194 | 203 | 68 | 145 | 140 |
| **Bilirubin umol/l** | 103 | 265 | 84 | 369 | 300 | 397 | 378 | 379 | 392 | 312 | 297 | 298 |
| **Albumin g/l** | 27 | 33 | 24 | 26 | 18 | 21 | 26 | 23 | 28 | 26 | 26 | 25.3 |
| **AST iU/l** | 117 | 72 | 126 | 79 | 149 | 57 | 249 | 98 | 105 | 199 | 89 | 122 |
| **INR** | 1.9 | 2.6 | 2.1 | 1.8 | 1.7 | 3.2 | 1.3 | 2.1 | 1.6 | 2.2 | 2.1 | 2.05 |
| **Maddrey's DF** | 45.1 | 80.8 | 50.5 | 55.2 | 48.4 | 109 | 38.7 | 74.1 | 43.2 | 68.8 | 66.6 | 61.9 |
| **MELD Score** | 20 | 29 | 28 | 26 | 24 | 34 | 30 | 27 | 25 | 28 | 27 | 27.1 |
| **CP Score** | 13 | 13 | 13 | 12 | 14 | 15 | 14 | 14 | 12 | 13 | 11 | 13.1 |
| **Septic (y/n)** | Y | N | N | Y | Y | Y | Y | Y | Y | Y | Y | Y |

**Supplemental Table 2. Clinical characteristics of patients with acute alcoholic hepatitis.** WCC – white cell count. AST – aspartate aminotransferase; INR – international normalised ratio; DF – discriminant function; MELD – Model for end-stage liver disease, CP – Child-Pugh.
